# The role of hypoxia-inducible factor-1α in zinc oxide nanoparticle-induced nephrotoxicity in vitro and in vivo

**DOI:** 10.1186/s12989-016-0163-3

**Published:** 2016-09-27

**Authors:** Yuh-Feng Lin, I-Jen Chiu, Fong-Yu Cheng, Yu-Hsuan Lee, Ying-Jan Wang, Yung-Ho Hsu, Hui-Wen Chiu

**Affiliations:** 1 Division of Nephrology, Department of Internal Medicine, Shuang Ho Hospital, Taipei Medical University, Taipei, Taiwan; 2 Graduate Institute of Clinical Medicine, College of Medicine, Taipei Medical University, 250 Wuxing Street, 110 Taipei, Taiwan; 3 Institute of Oral Medicine, National Cheng Kung University, Tainan, Taiwan; 4 Department of Environmental and Occupational Health, College of Medicine, National Cheng Kung University, Tainan, Taiwan; 5 Department of Biomedical Informatics, Asia University, Taichung, Taiwan; 6 Department of Medical Research, China Medical University Hospital, China Medical University, Taichung, Taiwan; 7 Department of Internal Medicine, School of Medicine, College of Medicine, Taipei Medical University, Taipei, Taiwan

**Keywords:** Hypoxia-inducible factor-1α, Zinc oxide nanoparticles, Nephrotoxicity, Autophagy, Apoptosis

## Abstract

**Background:**

Zinc oxide nanoparticles (ZnO NPs) are used in an increasing number of products, including rubber manufacture, cosmetics, pigments, food additives, medicine, chemical fibers and electronics. However, the molecular mechanisms underlying ZnO NP nephrotoxicity remain unclear. In this study, we evaluated the potential toxicity of ZnO NPs in kidney cells in vitro and in vivo.

**Results:**

We found that ZnO NPs were apparently engulfed by the HEK-293 human embryonic kidney cells and then induced reactive oxygen species (ROS) generation. Furthermore, exposure to ZnO NPs led to a reduction in cell viability and induction of apoptosis and autophagy. Interestingly, the ROS-induced hypoxia-inducible factor-1α (HIF-1α) signaling pathway was significantly increased following ZnO NPs exposure. Additionally, connective tissue growth factor (CTGF) and plasminogen activator inhibitor-1 (PAI-1), which are directly regulated by HIF-1 and are involved in the pathogenesis of kidney diseases, displayed significantly increased levels following ZnO NPs exposure in HEK-293 cells. HIF-1α knockdown resulted in significantly decreased levels of autophagy and increased cytotoxicity. Therefore, our results suggest that HIF-1α may have a protective role in adaptation to the toxicity of ZnO NPs in kidney cells. In an animal study, fluorescent ZnO NPs were clearly observed in the liver, lungs, kidneys, spleen and heart. ZnO NPs caused histopathological lesions in the kidney and increase in serum creatinine and blood urea nitrogen (BUN) which indicate possible renal possible damage. Moreover, ZnO NPs enhanced the HIF-1α signaling pathway, apoptosis and autophagy in mouse kidney tissues.

**Conclusions:**

ZnO NPs may cause nephrotoxicity, and the results demonstrate the importance of considering the toxicological hazards of ZnO NP production and application, especially for medicinal use.

**Electronic supplementary material:**

The online version of this article (doi:10.1186/s12989-016-0163-3) contains supplementary material, which is available to authorized users.

## Background

Hypoxia-inducible factor-1 (HIF-1) is a transcription factor that mediates adaptive responses to hypoxia at both the cellular and systemic levels [1]. HIF-1 is a heterodimer composed of the HIF-1α and HIF-1β subunit. It is regulated primarily through oxygen-dependent changes in the stability of the α subunit [2]. Hypoxic stimuli increase HIF-1α protein by inhibiting its degradation by the proteasome [3]. HIF-1 has been shown to regulate the expression of hundreds of target genes involved in angiogenesis, erythropoiesis, metabolism, apoptosis, autophagy, and other adaptive responses to hypoxia [4, 5]. Previous studies have demonstrated that reactive oxygen species (ROS) are involved in the oxygen-sensing mechanism. The exogenous application of H_2_O_2_ can induce HIF-1α under normoxic conditions and ROS scavengers can block hypoxic induction of HIF-1 [6]. In addition, HIF-1 is involved in the regulation of many biological processes that are related to kidney function under physiological and pathological conditions. In fully developed kidneys, HIF-1α is expressed in most cell types [7]. Factors that are directly regulated by HIF and are involved in the pathogenesis of acute and chronic kidney diseases include heme oxygenase-1 (HO-1), vascular endothelial growth factor (VEGF), plasminogen activator inhibitor-1 (PAI-1), tissue inhibitor of metalloproteinase-1 (TIMP-1), connective tissue growth factor (CTGF), erythropoietin, Wilms’ tumor suppressor (WT-1), and others [8]. An increase in HIF-1α in human and rat diseases, including polycystic kidneys and polycystic livers, has been reported [9, 10].

Zinc oxide nanoparticles (ZnO NPs) have become increasingly common in electronics, catalysts, clothing, paints and sunscreens [11, 12]. Current interest in ZnO NPs is focused on their medicinal use and biological applications, including as a biosensor and for drug delivery [13]. However, these applications increase human and environmental exposure and the potential risk for toxicity [14]. NPs can enter the human body via different routes such as inhalation, ingestion and injection [15]. They can then translocate to the blood, causing adverse biological reactions in several organs. To increase the potential of nanomedicine in ZnO NPs, full attention is needed to safety and toxicological issues. Because injection of ZnO NPs are brought intentionally into the human body, the concentration of ZnO NPs is higher than inhalation and ingestion in environment [16]. Furthermore, the kidney is particularly susceptible to xenobiotics because of its high blood supply and its ability to concentrate toxins [17, 18]. ZnO NPs significantly decreased the total renal total glutathione level compared with control values, which indicates functional damage to kidney tissues [18]. Another recent study concluded that ZnO NPs can disturb energy metabolism and impair mitochondria and cell membrane in rat kidneys [19]. Huang et al. indicated that titanium NP inhalation might induce renal fibrosis through a ROS/reactive nitrogen species- mediated HIF-1α-upregulated TGF-β signaling pathway [20]. Therefore, ZnO NPs may induce nephrotoxicity. However, the underlying molecular mechanisms are unclear.

Autophagy is a tightly regulated intracellular bulk degradation and recycling system that plays important roles in cellular homeostasis [21]. Previous studies have demonstrated that upregulation of autophagy in the kidney proximal tubules was observed in several experimental acute kidney injury (AKI) models [22, 23]. Ischemia-reperfusion (I/R) injury increased predominantly the number of autophagic vesicles in proximal tubules [24]. Other groups have shown that autophagy is protective against cisplatin-induced AKI [25, 26]. Autophagy is essential for scavenging damaged organs in cells. Therefore, autophagy plays a protective role during kidney disease [21]. In addition, autophagy has been described as a HIF-1α-dependent adaptive response [27]. In some disease models, HIF signaling results in increased levels of a BH3-only protein—Bcl-2/E1B 19 kDa-interacting protein 3 (BNIP3), a protein that protects against cell death during activation of autophagy [28]. However, there are few published articles describing the relationship between autophagy and ZnO NPs [29, 30]. Collectively, the previously described reports demonstrate that exposure to ZnO NPs may affect renal cells. The objective of the present study was to investigate the effect of ZnO NPs on autophagy and apoptosis in kidney cells in vitro and in vivo. Furthermore, we examined the interplay between autophagy and HIF-1α in kidney cells exposed to ZnO NPs.

## Methods

### Cell culture and co-incubation with ZnO NPs

The human embryonic kidney cell line HEK-293 (ATCC CRL-1573) was obtained from the American Type Culture Collection (ATCC). Cells were maintained under standard growth conditions in a humidified incubator in Eagle’s minimum essential medium (MEM), 10 % fetal bovine serum, 100 U/ml penicillin, 100 μg/ml streptomycin 0.1 mM non-essential amino acids and 1.0 mM sodium pyruvate (Gibco BRL, Grand Island, NY) at 37 °C and 5 % CO_2_. Exponentially growing cells were detached with 0.05 % trypsin-EDTA (Gibco BRL, Grand Island, NY) in MEM medium. ZnO NP solutions were freshly prepared from stock solutions and sonicated for 5 min before addition to cell cultures.

### Preparation and physicochemical characterization of ZnO NPs

First, ZnO NPs were obtained by as previously reported [31]. Zinc acetate dihydrate (Zn(CH_3_COO)_2_(H_2_O)_2_; 147 mg) was dissolved in 6.25 ml of methanol and a potassium hydroxide (KOH) solution was prepared by dissolving 74 mg of KOH in 3.25 ml of methanol. Then, the KOH solution was added dropwise into the zinc acetate solution with vigorous stirring after heating the zinc acetate solution to 60 °C. After 1.5 h, the solution become turbid and precipitates were slowly produced after the solution was allowed to stand at room temperature for another 2 h. The precipitates (ZnO NPs) were collected by centrifugation at 10,000 rpm for 10 min and were washed twice with methanol. The precipitates were dispersed in 10 ml ethanol. Then, 50 μL of (3-aminopropyl) triethoxysilane (APTES), 100 μl of deionized water, and 10 μl of 25 % wt ammonia aqueous solution were added to the ZnO NP solution. The mixed solution was stirred at room temperature for 20 h. The solution was centrifuged at 10,000 rpm for 15 min, and the supernatant was discarded. The precipitates (NH_2_-ZnO@SiO_2_ NPs) were washed twice with ethanol and re-dispersed in ethanol or deionized water for further use.

The average hydrodynamic size and polydispersity index (PDI) of the ZnO NPs were determined by dynamic laser scattering (Delsa™ Nano C, Beckman Coulter, Inc., USA). This instrument is capable of measuring particles ranging from 0.6 nm to 7 μm. The zeta potential of the ZnO NPs was measured by the Zetasizer Nano ZS90 (Malvern Instruments Ltd,, UK). The size measurements were performed on dilute ZnO NP suspensions in aqueous solution and MEM (including 10 % FBS). Characterization of the ZnO NPs was performed using transmission electron microscopy (TEM) (JEOL Co., MA, USA). ZnO NPs were examined after suspension in aqueous solution and subsequent deposition onto copper-coated carbon grids.

Zinc release from the particle suspension was measured by ICP-AES (Jobin Yvon JY138 spectroanalyzer, Horiba Jobin Yvon, Inc., USA). Samples were prepared by diluting the stock particle suspension in complete culture medium at a concentration of 20 μg/ml ZnO NPs for 24 h. After incubation, aliquots of the particle suspensions were centrifuged. Then, the samples were microwave digested in reverse aqua regia (1:3 HCl:HNO_3_) and zinc concentration was analyzed by ICP-AES. Results were expressed as the percentage of the tested suspension.

### Cell viability assay

The cellular viability was assessed by the MTS assay, which measures at the reduction of (3-(4,5-dimethylthiazol-2-yl)- 5-(3-carboxymethoxyphenyl)-2-(4-sulfophenyl)-2*H*-tetrazolium (MTS) to formazan in viable cells. Briefly, cells were plated onto 96-well plates (Thermo, MA, USA). After incubation with the indicated dose of ZnO NPs for various lengths of time at 37 °C, formazan absorbance was measured at 490 nm. The mean absorbance of non-exposed cells was the reference value for calculating 100 % cellular viability.

### ZnO NP uptake using fluorescence confocal microscopy and flow cytometry

We plated cells onto 6-well plates with a glass coverslip per well. After ZnO NP exposure, cells were fixed with 4 % paraformaldehyde. After three washes in PBS, the cells were stained with 4′-6-diamidino-2-phenylindole (DAPI) (Sigma, MO, USA). Fluorescence confocal images were taken using a confocal microscope (Carl Zeiss LSM 780, Instrument Development Center, NCKU). The uptake of particles into cells was also analyzed by flow cytometry (Becton Dickinson, USA). The side scatter (SSC) data were analyzed using CELLQuest™ software (Becton Dickinson). Ten thousand cells were acquired for each measurement.

### ROS production measurement

To measure ROS generation, a fluorometric assay was carried out using the intracellular oxidation of 2,7-dichlorofluorescein diacetate (DCFH-DA, Sigma, MO, USA). The cells were treated with different concentrations of the ZnO NPs for 2, 4 or 6 h and were then incubated with 10 μM DCFH-DA for 30 min. After washing with PBS, DCFH fluorescence of the cells from each well was measured in a fluorescence microplate reader (Thermo, MA, USA) at an excitation wavelength of 485 nm and emission at 530 nm. The intensity of fluorescence reflects the extent of oxidative stress.

### Annexin V and propidium iodide (PI) staining assay

Cells were trypsinized, washed with PBS and centrifuged at 2000 rpm for 5 min. Cells were resuspended in 100 μl of 1 × Annexin V binding buffer (10 mM HEPES (pH 7.4), 0.14 M NaCl and 2.5 mM CaCl_2_) that contained 2 μl of Annexin V-FITC (Calbiochem, CA, USA) alone or in combination with 10 μl of PI (50 μg/ml) and were incubated in the dark at room temperature for 15 min. The 1× binding buffer (400 μl) was added to stop the reaction, and staining was analyzed by FACScan flow cytometry (Becton Dickinson, USA).

### Cell staining with acridine orange for detection of autophagy

Cell staining with acridine orange (AO) (Sigma Chemical Co., USA) was performed according to published procedures [32].

### Immunofluorescence microscopy

The cells were cultured on coverslips. After ZnO NP treatment, the cells were fixed in 4 % paraformaldehyde and blocked with 1 % BSA for 30 min. This was followed by incubation with a specific antibody against microtubule-associated protein light chain 3 (LC3) (MBL, Japan) for 1 h. After washing, the cells were labeled with a DyLight™ 488-conjugated AffiniPure goat anti-rabbit IgG (Jackson ImmunoResearch Laboratories, PA, USA) for 1 h and stained with DAPI. Finally, the cells were washed in PBS, coverslipped, and examined with a fluorescence microscope or confocal microscope (Carl Zeiss LSM780, Instrument Development Center, NCKU).

### Western blot analysis

Total cellular protein lysates were prepared by harvesting cells in protein extraction buffer for 1 h at 4 °C as described previously [32]. GAPDH was used as the protein loading control. Anti-PAI-1, anti-Bax and anti-Beclin 1 were obtained from Cell Signaling Technology (Ipswich, MA, USA); anti-HIF-1α was obtained from BD Transduction Laboratories (San Diego, CA, USA); anti-GAPDH was obtained from Abcam (Cambridge, MA, USA); anti-LC3 and anti-CTGF were obtained from Abgent (San Diego, CA, USA); anti-p62/SQSTM1 was obtained from MBL (Nagoya, Japan); anti-pro-caspase 3 and anti-cleaved-caspase 3 were obtained from Epitomics (Burlingame, CA, USA).

### RNA interference (RNAi)

We used Arrest-In Transfection Reagent (Thermo, MA, USA) to transfect cells according to the manufacturer’s protocol. HIF-1α siRNA (ID: s6541) was obtained from ThermoFisher Scientific (Waltham, MA, USA).

### Biodistribution

First, to prepare red-NIR700-ZnO NPs, fluorescent red-NIR700 (purchased from Sigma-Aldrich, 61059, λ_ex_ 672 nm; λ_em_ ~735 nm) was used because red-NIR700 has an N-hydroxysuccinimide (NHS) group and can form a covalent bond with a primary amine. of R6G-isothiocyanates (0.2 ml of 0.01 mM) and NH_2_-ZnO@SiO_2_ NPs (2 ml of 200 μg/ml) were mixed together and stirred at room temperature for 4 h. Red-NIR700-ZnO@SiO_2_ NPs were collected by centrifugation at 10,000 rpm for 15 min, and the supernatant was removed. The isolated products were washed twice with ethanol and re-dispersed in deionized water.

All experiments using mice were performed according to the guidelines of our institutes (the Guide for Care and Use of Laboratory Animals, National Cheng Kung University and Taipei Medical University). Female BALB/c mice (8 weeks old) were acquired from the animal center of the National Cheng Kung University Medical College. The animals were housed 5 per cage at 24 ± 2 °C with 50 ± 10 % relative humidity and subjected to a 12-h light/12-h dark cycle. BALB/c mice were i.p. injected with red-NIR700-ZnO NPs (a dose of 10 mg/kg) and then were sacrificed at 6 h after injection. Various organs were collected, and fluorescence imaging was conducted using an IVIS 200 imaging system coupled to a data acquisition computer running Living Image Software (Xenogen).

### In vivo experiment protocol

Female BALB/c mice (8 weeks old) were acquired from the BioLASCO Experimental Animal Center (Taiwan). The animals were housed 5 per cage at 24 ± 2 °C with 50 ± 10 % relative humidity and subjected to a 12-h light/12-h dark cycle. The animals were acclimatized for 1 week prior to the start of the experiments. Mice were fed a Purina chow diet with water ad libitum. Mice were randomly divided into four groups (5 animals/group): ZnO NPs once per week for four weeks. Varying concentrations of ZnO NPs were suspended in MQ water. ZnO NP solutions were freshly prepared from stock solutions and sonicated for 5 min before intraperitoneal (i.p.) or intravenous (i.v.) injection. Mice were sacrificed via CO_2_ exposure. After euthanasia, the kidney tissues of mice were formalin-fixed and paraffin-embedded for immunohistochemistry.

### Inductively coupled plasma atomic emission spectroscopy (ICP-AES)

To evaluate zinc levels in the different tissues, samples from the lung, liver, kidney, spleen and heart were obtained and weighed. The samples were microwave digested in reverse aqua regia (1:3 HCl:HNO_3_) and analyzed for zinc concentrations by ICP-AES (Jobin Yvon JY138 spectroanalyzer, Horiba Jobin Yvon, Inc., USA).

### Biochemical tests

Whole blood samples from treated mice were collected by intracardiac puncture and centrifuged at 2000 × g for 20 min to separate the serum. Biochemical evaluations included glutamate oxaloacetate transaminase (GOT) activity, glutamate pyruvate transaminase (GPT) activity, blood urea nitrogen (BUN) levels and creatinine levels. The urinary creatinine concentrations were detected by Parameter Creatinine assay kit (R&D Systems, MN, USA). Creatinine clearance (CCr) was calculated by the standard formula CCr = (U × V)/S, where U is the creatinine concentration in urine (mg/dL), V is urine flow rate (μl/min), and S is the creatinine concentration in serum (mg/dL).

### Histopathological and immunohistochemical (IHC) staining analysis

Paraffin-embedded tissue sections (2 μm) were dried, deparaffinized, and rehydrated. Following microwave pretreatment in citrate buffer (pH 6.0; for antigen retrieval), the slides were immersed in 3 % hydrogen peroxide for 20 min to block the activity of endogenous peroxidase. The histopathological observations were interpreted by staining tissue sections with hematoxylin and eosin. Tissue sections were immunohistochemically incubated overnight at 4 °C with the anti-LC3 (MBL, Japan), anti-HIF-1α (BD Transduction Laboratories, USA) or anti-cleaved-caspase 3 (Epitomics, USA) antibodies. The sections were then incubated with a secondary antibody for 1 h at room temperature, and the slides were developed using the STARR TREK Universal HRP detection kit (Biocare Medical, Concord, CA). Finally, the slides were counterstained using hematoxylin.

### Statistical analysis

Data are expressed as the mean ± SD. Statistical significance was determined using Student’s *t*-test for comparison between the means or one-way analysis of variance with a post-hoc Dunnett’s test. Differences were considered significant when *p* <0.05.

## Results

### ZnO NP characterization

**Fig. 1:**
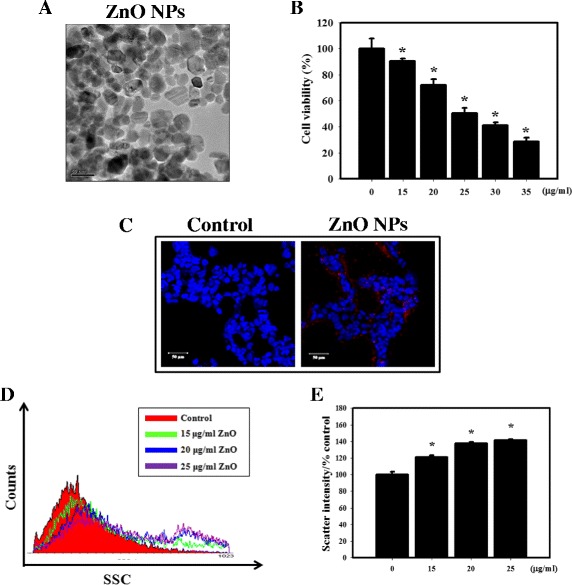
Cellular uptake and cytotoxicity of ZnO NPs by HEK-293 cells. **a** TEM analysis of ZnO NP morphology. ZnO NPs were mainly spherical in shape. The scale bar represents 100 nm. **b** Cell viability was measured using the MTS assay. ZnO NPs decreased the viability of HEK-293 cells in a concentration-dependent manner. The cells were treated with 0, 15, 20, 25, 30 or 35 μg/ml ZnO NPs for 24 h. All of the MTS values were normalized to the control values (no particle exposure), which were regarded as 100 % cell viability. *p < 0.05 versus control. **c** Uptake of ZnO NPs detected by fluorescence confocal microscopy. ZnO NPs are shown in red and DAPI (*blue*) is a nuclei-specific marker. The cells were treated with 20 μg/ml ZnO NPs for 8 h. **d**, The results of the SSC data and flow cytometry demonstrated that ZnO NPs were apparently engulfed by HEK-293 cells. **e** Quantification of the scatter intensity in HEK-293 cells with ZnO NP treatment. The cells were treated with ZnO NPs at 0, 15, 20, or 25 μg/ml for 24 h. The data are presented as the mean ± standard deviation of three independent experiments

To assess the physical characteristics of ZnO NPs, several parameters were measured. The detailed results of the physical characterization after suspension in cell culture medium or water are presented in Table 1. ZnO NPs had a hydrodynamic diameter of 47.8 nm in water and 70.5 nm in MEM. The zeta potential of ZnO NPs in water was positive (+36.7), which ZnO NPs in MEM had negative charges (−8.09). As previously reported, all of the NPs have negatively charged surfaces in cell culture medium due to the formation of a corona of negatively charged proteins [33]. The polydispersity index (PDI) indicates the dispersion stability and solubility of NPs in water or medium, and a PDI value lower than 0.2 is associated with a high homogeneity of the particle population [34]. The values for ZnO NPs in water and MEM were 0.128 and 0.230, respectively. Furthermore, dissolution of NPs is an important property that influences their mode of action (e.g. antimicrobial properties, toxicity, medicinal applications and environmental impact) [35]. In our case, the release of zinc ions after 24 h of incubation was low, <1 % of the initial concentrations for ZnO NPs in water and MEM. In addition, the TEM image suggests that the particles are polydispersed and are mostly spherical in shape (Fig. 1a).

**Table 1:** Physical characteristics of ZnO NPs

	Hydrodynamic diameter (nm)		PDI^a^		Zeta potential (mV)		Dissolution (μg/ml)^b^	
	Water	MEM	Water	MEM	Water	MEM	Water	MEM
ZnO	47.8	70.5	0.128	0.230	+36.7	−8.09	0.12	0.18

^a^PDI is the polydispersity index

^b^Zinc ions released from 20 μg/ml ZnO NPs for 24 h measured by ICP-AES

### Cytotoxic effects, cellular uptake and the HIF-1α signaling pathway in HEK-293 cells treated with ZnO NPs

To assess the cytotoxic effects of ZnO NPs in kidney cells, we used the MTS assay (Fig. 1b). Our results demonstrated that ZnO NPs reduced the viability of HEK-293 cells in a concentration-dependent manner. Next, to evaluate the entry of the ZnO NPs into the cells, we used fluorescent ZnO NPs (Fig. 1c). Confocal microscopy analysis showed that exposure to ZnO NPs increased the fluorescence intensity of HEK-293 cells. The side scatter (SSC) intensity analyzed by flow cytometry also confirmed that the ZnO NPs were apparently engulfed by the HEK-293 cells in a concentration-dependent manner (Figs. 1d-f).

**Fig. 2:**
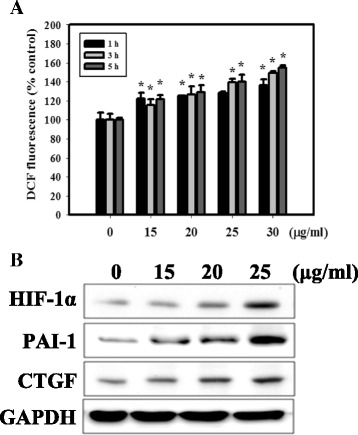
Effects of ZnO NPs on cellular ROS and the expression of HIF-1α-related proteins in HEK-293 cells. **a** ROS generation in HEK-293 cells treated with ZnO NPs for 1, 3 or 5 h and with DCFH-DA for an additional 30 min. The fluorescence in the cells was immediately analyzed using a fluorescence microplate reader. **b** Western blotting for HIF-1α, PAI-1 and CTGF in HEK-293 cells. The cells were treated with ZnO NPs for 24 h

ZnO NPs may cause oxidative stress resulting in lipid peroxidation, cell membrane damage and, ultimately, cell death or apoptosis in macrophages and human cells [36]. We determined the intracellular ROS level by DCFH-DA (Fig. 2a). The results showed that ZnO NPs stimulated ROS formation in cells in a concentration-dependent manner. It has been reported that ROS induce HIF-1α activation [37]. HIF-1α is involved in the pathogenesis of kidney diseases, as are PAI-1 and CTGF [8]. As shown in Fig. 2b, the expression of HIF-1α, PAI-1 and CTGF significantly increased following ZnO NP exposure.

### Measurement of apoptosis and autophagy in HEK-293 cells treated with ZnO NPs

**Fig. 3:**
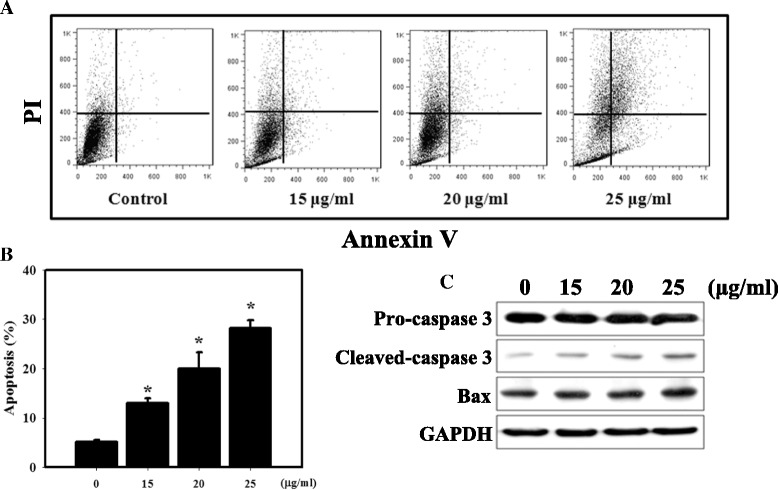
Measurement of apoptosis in HEK-293 cells treated with ZnO NPs. **a** Annexin V/PI staining in HEK-293 cells treated with ZnO NPs. The induction of apoptosis and necrosis was determined by flow cytometric analysis of Annexin V and PI staining. **b** Quantification of apoptotic cells with Annexin V-stained cells using flow cytometry. HEK-293 cells treated with different concentrations of ZnO NPs were assessed using Annexin V/PI staining. Cells were incubated with 0–25 μg/ml ZnO NPs for 24 h. *p < 0.05 versus control. The data are presented as the mean ± standard deviation of three independent experiments. **c** Western blotting for procaspase-3, cleaved-caspase-3 and Bax in HEK-293 cells. Cells were treated with 0–25 μg/ml ZnO NPs for 24 h

**Fig. 4:**
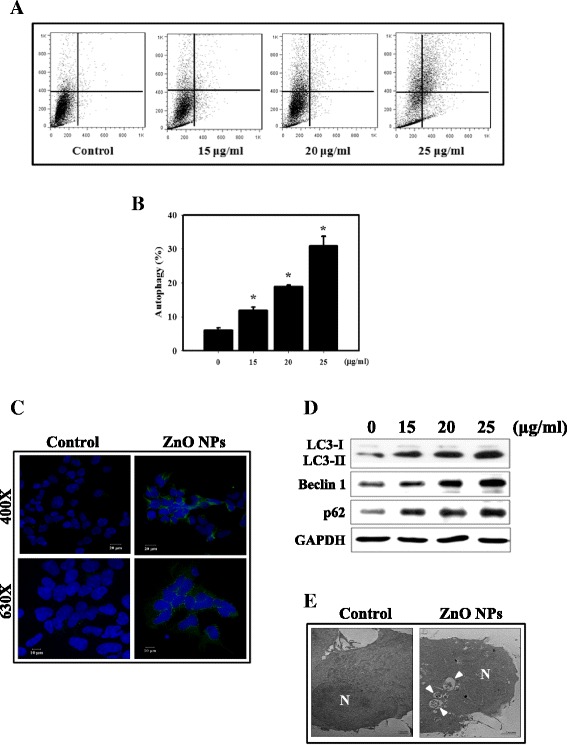
Measurement of autophagy in HEK-293 cells treated with ZnO NPs. **a** Development of AVOs in HEK-293 cells. Detection of green and red fluorescence in AO-stained cells using flow cytometry. **b** Quantification of AVOs treated with ZnO NPs with AO. Cells were incubated with 0–25 μg/ml ZnO NPs for 24 h. *p < 0.05 versus control. The data are presented as the mean ± standard deviation of three independent experiments. **c** Immunofluorescence staining of the LC3 protein in HEK-293 cells treated with 20 μg/ml ZnO NPs for 24 h. **d** Western blotting for LC3-I, LC3-II, Beclin 1 and p62 in HEK-293 cells. Cells were treated with 0–25 μg/ml ZnO NPs for 24 h. **e** Ultrastructural changes observed in HEK-293 cells after ZnO NP treatment. Cells were treated with medium alone (untreated) or 20 μg/ml ZnO NPs for 24 h. The *white arrowheads* indicate the autophagic vacuoles and autolysosomes. N, nucleus

Apoptosis in HEK-293 cells was measured by flow cytometry following Annexin V and PI staining (Fig. 3a and b). We observed a substantial increase in percentage of apoptotic cells following treatment with ZnO NPs. The expression of apoptosis-related proteins (cleaved-caspase 3 and Bax) was examined by western blotting analysis (Fig. 3c). The cleaved-caspase 3 and Bax protein levels increased following ZnO NP treatment compared with the control. Furthermore, we analyzed the type II programmed cell death, autophagy, which is characterized by the presence of acidic vesicular organelles (AVOs) in the cell cytoplasm. AVO formation was detected and measured by vital staining with acridine orange (AO) [38]. We found a significantly increased amount of AO-positive cells in the ZnO NP treatment group compared to the control (Fig. 4a and b). We detected autophagosomes in ZnO NP-treated cells using a green fluorescence-labeled LC3 immunofluorescence assay (Fig. 4c). The results showed that the number of vacuoles (green dots inside cells) increased conspicuously. Meanwhile, we observed increased expression of the autophagy-related proteins LC3-II, Beclin 1 and p62 following ZnO NP treatment (Fig. 4d). Next, we performed TEM analysis to analyze the ultrastructures of the HEK-293 cells treated with ZnO NPs (Fig. 4e). In the cytoplasm of the cells treated with ZnO NPs, we observed a large number of autophagic vacuoles.

### The HIF-1α signaling pathway is involved in the ZnO NP-induced autophagy in HEK-293 cells

**Fig. 5:**
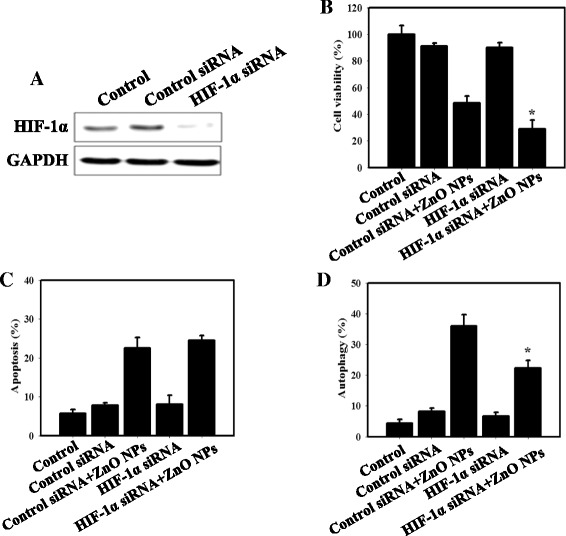
HIF-1α knockdown by siRNA affects the cytotoxicity and autophagy of HEK-293 cells. **a** Transfection efficacy was verified by western blot analysis. HIF-1α protein expression in HEK-293 cells transfected with control or HIF-1α siRNA for 24 h. **b** Cell viability in the absence or presence of HIF-1α siRNA in HEK-293 cells. **c** Quantification of apoptotic cells with Annexin V-stained cells using flow cytometry. **d** Quantification of AVOs with AO-stained cells transfected with control or HIF-1α siRNA using flow cytometry. The cells were transfected with control or HIF-1α siRNA for 24 h and treated with medium alone or 20 μg/ml ZnO NPs for 24 h. *p < 0.05, control siRNA + ZnO NPs versus HIF-1α siRNA + ZnO NPs

To further define the role of HIF-1α, we silenced HIF-1α expression using HIF-1α siRNA in HEK-293 cells. The expression of the HIF-1α proteins was markedly decreased in the cells treated with the HIF-1α siRNA compared with the control siRNA (Fig. 5a). The viability of the cells after siRNA transfection and ZnO NP treatment was determined by MTS assays. As shown in Fig. 5b, transfection with HIF-1α siRNA significantly enhanced the cytotoxicity of ZnO NPs in HEK-293 cells. Furthermore, we used HIF-1α siRNA to determine whether inhibition of HIF-1α alters ZnO NP treatment-induced apoptosis and autophagy. The results indicated that ZnO NP treatment with HIF-1α siRNA let to a significant decrease in autophagic cells compared to ZnO NPs alone (Fig. 5d). However, HIF-1α siRNA alone showed no noticeable change in apoptotic cells (Fig. 5c). These results suggest that HIF-1α-knockdown enhances the ZnO NP-induced cytotoxicity and suppresses the ZnO NP-induced autophagy.

### In vivo biodistribution and nephrotoxicity in ZnO NP-treated mice

**Fig. 6:**
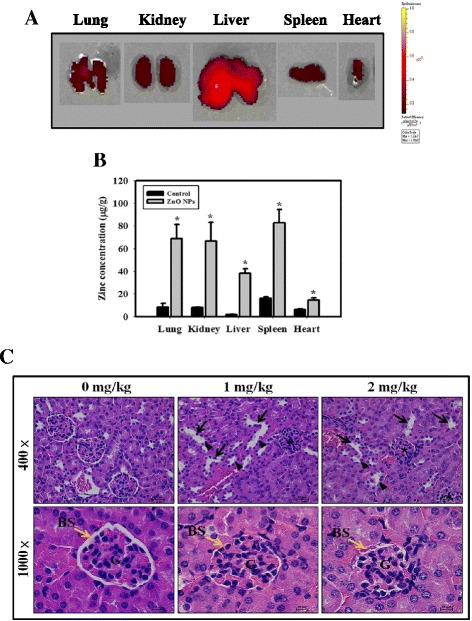
In vivo biodistribution of ZnO NPs and histopathological analysis of kidneys after i.p. injection of ZnO NPs. **a** The images of liver, lungs, kidneys, spleen and heart under NIR illumination clearly demonstrate the presence of ZnO NPs in these organs. BALB/c mice were i.p. injected with red-NIR700-ZnO NPs (a dose of 10 mg/kg) and were sacrificed 6 h after injection. Various organs were collected, and fluorescence imaging was conducted using an IVIS 200 imaging system. **b** Concentration of ZnO NPs in tissues. The mice were i.p. injected with ZnO NPs (a dose of 10 mg/kg) and were sacrificed 6 h after injection. The concentrations of Zn in the lung, kidney, liver, spleen and heart were evaluated using an ICP-AES. *p < 0.05 versus control. **c** Histopathological kidney lesions were found in ZnO NP-treated mice. Tissue sections were stained with H&E and observed microscopically. The *black arrows* indicate tubular dilatation. The *black arrowheads* indicate the loss of brush borders and flattened tubular epithelium. The *asterisks* indicate the reduction of Bowman’s space and the increase in cellularity in glomeruli. BS, bowman space; G, glomerulus

In vivo biodistribution of ZnO NPs can provide essential information on ZnO NP behavior post administration. We mimicked the medicinal use of ZnO NPs and thus used i.p. or i.v. injection for administration of ZnO NPs. To achieve this aim with high sensitivity, red-NIR700, a commonly used NIR fluorescent dye, was used with ZnO NPs to elucidate ZnO NP biodistribution in vivo. BALB/c mice were i.p. injected with red-NIR700-labeled ZnO NPs (at a dose of 10 mg/kg) and then sacrificed after 6 h. Our results showed that ZnO NPs can accumulate in the liver, lungs, kidneys, spleen and heart (Fig. 6a). In addition, the concentrations of Zn were evaluated using an ICP-AES (Fig. 6b). The Zn concentration in the lung, kidney, liver, spleen and heart showed a significant increase in comparison with the control groups. To further investigate the nephrotoxicity of ZnO NPs, the mice were i.p. or i.v. injected with varying concentrations of ZnO NPs. For the serum biochemical analysis, the activities of GOT, GPT, creatinine and BUN were significantly increased in the ZnO NP-treated mouse groups (Table 2 and Additional file 1: Table S1). ZnO NP treatments also decreased CCr levels (Additional file 1: Figure S1). Furthermore, the kidneys were sliced and stained with H&E. The histopathological lesions of the kidneys showed tubular dilatation, loss of brush borders and flattening of tubular epithelium, reduced Bowman’s space and increased cellularity in the glomeruli of ZnO NP-treated mice (Fig. 6c and Additional file 1: Figure S2).

**Table 2:** Biochemical tests including BUN, creatinine, GOT and GPT after i.p. injection of ZnO NPs

Item/Group	0 mg/kg	1 mg/kg	2 mg/kg	4 mg/kg
BUN (mg/dL)	18.76 ± 3.75	23.46 ± 3.06	19.38 ± 0.57	23.40 ± 1.67*
Creatinine (mg/dL)	0.26 ± 0.02	0.33 ± 0.02*	0.33 ± 0.03*	0.35 ± 0.04*
GOT (U/L)	114.20 ± 22.13	116.40 ± 17.77	153.20 ± 27.40*	168.60 ± 29.53*
GPT (U/L)	31.80 ± 0.84	32.00 ± 3.32	34.40 ± 2.88	36.40 ± 1.52*

*p < 0.05, ZnO NPs versus control

**Fig. 7:**
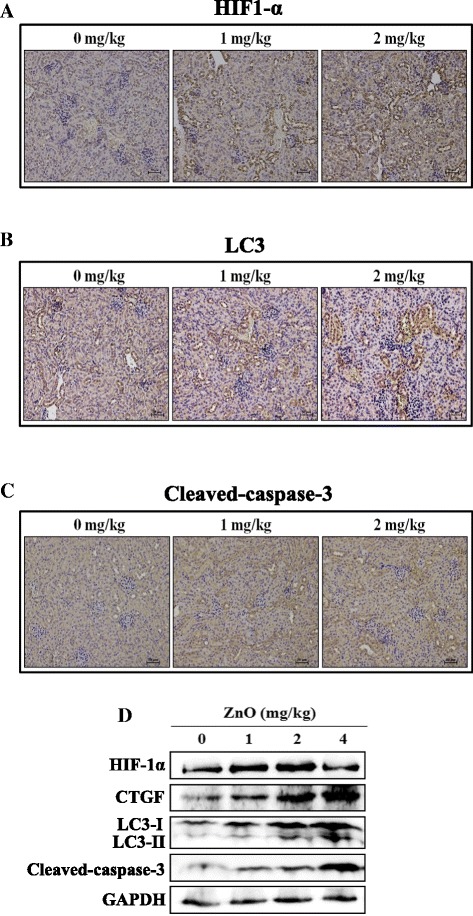
Protein expression in the kidney tissues using IHC staining and western blot analysis. IHC was used to determine the expression levels of HIF-1α (**a**), LC3 (**b**) and cleaved-caspase-3 (**c**). **d** Western blot analysis of protein expression in kidney tissues. The mice were i.p. injected with ZnO NPs

Next, HIF-1α, LC3 and cleaved-caspase-3 expression levels were examined in the kidney tissue using IHC staining (Fig. 7a-c). Significant increases in kidney expression of HIF-1α, LC3 and cleaved-caspase-3 were observed in the ZnO NP treatment group compared with the control group. In addition, proteins extracted from the kidney tissues were assayed by western blotting (Fig. 7d). We found that HIF-1α, CTGF, LC3-II and cleaved-caspase-3 protein levels were increased in the ZnO NP treatment group. Thus, ZnO NPs could cause renal histopathological lesions and regulate the HIF-1α signaling pathway in the kidney.

## Discussion

Many studies have examined the side effects of NPs, especially ZnO NPs, but unfortunately, there are few published articles describing the relationship between ZnO NPs and nephrotoxicity. Beckett et al. indicated that inhalation of ZnO fumes at relatively high dose (500 μg/m^3^) for 2 h in an occupational setting can cause metal fume fever (fatigue, chills, fever, myalgias, cough, dyspnea, leukocytosis, metallic taste and salivation) [39]. In healthy skin, the epidermis provides excellent protection against ZnO NPs spread to the dermis [40]. However, few human health studies are available on ZnO exposure. Previous research has shown that ZnO NPs can cause hepatic injury in mouse model [41]. In the present study, significant increases in GOT and GPT were found in all mice exposed to ZnO NPs (Table 2 and Additional file 1: Table S1). It is likely that renal exposure to NPs would be commensurate with the organ’s key role in the excretion of xenobiotics. The results from this study demonstrated that ZnO NPs had a severe renal toxicological effect and increased of blood biochemical markers (BUN and creatinine) which indicate possible renal damage (Fig. 6c, Additional file 1: Figure S2, Table 2 and Additional file 1: Table S1). ZnO NPs have been shown to induce cyto- and genotoxicity in kidney epithelial cells [42]. Our in vitro studies revealed that ZnO NPs were cytotoxic to the human kidney cell line HEK-293, possibly due to ROS generation (Figs. 1b and 2a). Furthermore, the uptake of NPs by cells is an important factor in their toxicity. Inhibition of cellular uptake of ZnO NPs would largely reduce the cytotoxicity [43]. The results of the present study indicated that ZnO NPs entered into cells in a concentration-dependent manner (Fig. 1c-f). In addition, ZnO NPs increased apoptosis in HEK-293 cells and in the kidneys of a mouse model (Figs. 3 and 7). The cellular uptake ROS generation and apoptosis may be the intrinsic reasons for the high toxicity of ZnO NPs. Previous studies have demonstrated that fluorescence labeling NPs had a small but significant reduction in the level of cytotoxicity and led to a minor increase in particle aggregation when compared against the non-labeled particles. A possible explanation is that fluorescence labeling affects the zeta potential of the particle, but not to the extent that it changes all the available NH_2_ groups [33]. In the present study, we not only utilized fluorescence labeling ZnO NPs but also used ZnO NPs without fluorescent dyes by ICP-AES in order to study in vivo biodistribution of ZnO NPs (Fig. 6a and b).

Autophagy is a complex catabolic pathway and a highly dynamic quality control mechanism to recycle cellular components, eliminate aberrant materials and ultimately maintain cellular homeostasis. Autophagy can be stimulated by different types of microorganisms, such as bacteria, viruses, or parasites. Because NPs are similar in size to some microorganisms, it is possible that autophagy is also activated upon internalization of NPs [44, 45], most likely as a protective response to what is perceived as foreign or toxic [46, 47]. Here we prepared ZnO NPs to examine the induction of autophagy. The formation of autophagosomes and autophagy-related proteins was significantly increased in ZnO NP-treated cells (Fig. 4). TEM analysis indicated that the autophagosome was formed from the surrounding proteins and damaged organelles destined for degradation (Fig. 4e). However, whether autophagy is protective for or cytotoxic to NPs remains controversial. Our previous study found that NPs increased autophagy due to ROS generation and endoplasmic reticulum (ER) stress [44]. Other recent studies including ours, concluded that exposure to NPs is a potential source of oxidative stress which leads to the induction of ROS, apoptosis and autophagy [36, 45]. Thus, we investigated the ROS generated induced by ZnO NPs. Our current findings showed higher ROS levels in the HEK-293 cells exposed to ZnO NPs than the controls (Fig. 2a).

Several studies have reported that the subunits of NADPH oxidase, which are one of the major sources of ROS, were related to HIF-1α expression in response to hypoxia in the renal interstitial cells or the A549 cells [48, 49]. Furthermore, recent evidence shows that NPs might induce renal fibrosis through a ROS- mediated HIF-1α signaling pathway [20]. In Fig. 2b, we demonstrated that HIF-1α expression was increased in cells treated with ZnO NPs. Additionally, CTGF and PAI-1, which are directly regulated by HIF and are related to the pathogenesis of kidney diseases, displayed significantly increased levels following ZnO NPs exposure in HEK-293 cells. In vivo significant increases in kidney-expressed HIF-1α and CTGF were observed in the ZnO NP treatment group compared with the control group (Fig. 7a and d). It is worth mentioning that HIF-1α induces autophagy through BNIP3. BNIP3 stimulates autophagy by preventing the inhibitory binding of Bcl-2 to Beclin-1, which frees Beclin-1 to participate in autophagosome formation [50]. In our current study, HIF-1α knockdown by HIF-1α siRNA revealed significantly decreased levels of autophagy and increased cytotoxicity (Fig. 5). Therefore, our results suggest that HIF-1 may have a protective role in adaptation to the toxicity of ZnO NPs in kidney cells.

## Conclusions

**Fig. 8:**
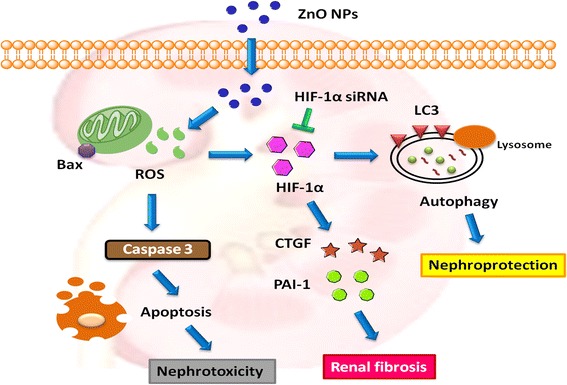
ZnO NPs pathways and effects in kidney cells. ZnO NPs induce apoptosis and the HIF-1α signaling pathway through ROS generation, eventually leading to nephrotoxicity. Furthermore, ZnO NP-induced autophagy may be mediated by the induction of the HIF-1α signaling pathway. Inhibition of HIF-1α by HIF-1α siRNA increases the cytotoxicity, indicating a protective role of HIF-1α. In addition, CTGF and PAI-1, which are directly regulated by HIF and are related to the renal fibrosis, are increased after ZnO NP treatment

In the present study, the results showed that ZnO NPs can induce apoptosis and autophagy, accompanied by ROS and the activation of the HIF-1α signaling pathway, eventually leading to nephrotoxicity (Fig. 8). Our findings suggest that ZnO NP-induced autophagy is associated with HIF-1α signaling, which may be an important mechanism and protective outcome of kidney diseases caused by ZnO NPs. In the animal study, the histological analysis of kidneys from ZnO NP-treated mice showed histopathological lesions. Furthermore, serum biochemical analysis showed that BUN, creatinine, GPT and GOT activities, which indicate possible renal and liver damage, were significantly elevated in ZnO NP-treated mice. Therefore, it is necessary for all researchers or patients who are regularly exposed to ZnO NPs to consider the toxicological hazards and institute appropriate safety measures.

## Additional file

Additional file 1:
**Table S1.** Biochemical tests including BUN, creatinine, GOT and GPT after i.v. injection of ZnO NPs. **Figure S1.** Measurements of the creatinine clearance (CCr) in BALB/c mice during i.p. and i.v. injection of ZnO NPs. Clearance was calculated based on urine volumes and serum and urine creatinine concentrations. **p* < 0.05 versus 0 mg/kg. **Figure S2.** Histopathological analysis of kidneys after i.v. injection of ZnO NPs (2 mg/kg). Tissue sections were stained with H&E and observed microscopically. The black arrows indicate tubular dilatation. The black arrowheads indicate the loss of brush borders and flattened tubular epithelium. The asterisks indicate the reduction of Bowman’s space and the increase in cellularity in glomeruli. BS, bowman space; G, glomerulus. (DOCX 1351 kb)

## Abbreviations

AKI: Acute kidney injury
AO: Acridine orange
AVOs: Acidic vesicular organelles
BUN: Blood urea nitrogen
CTGF: Connective tissue growth factor
ER: Endoplasmic reticulum
GOT: Glutamate oxaloacetate transaminase
GPT: Glutamate pyruvate transaminase
i.p.: Intraperitoneal
I/R: Ischemia-reperfusion
LC3: Microtubule-associated protein light chain 3
PAI-1: Plasminogen activator inhibitor-1
ROS: Reactive oxygen species
SSC: Side scatter
ZnO NPs: Zinc oxide nanoparticles
