# Supplementary material for: The role of hypoxia-inducible factor-1α in zinc oxide nanoparticle-induced nephrotoxicity in vitro and in vivo
Source: Part Fibre Toxicol. 2016 Sep 27;13:52. doi: 10.1186/s12989-016-0163-3 (PMC5037597; doi:10.1186/s12989-016-0163-3)
Supplement: Additional file 1: — Table S1. Biochemical tests including BUN, creatinine, GOT and GPT after i.v. injection of ZnO NPs. Figure S1. Measurements of the creatinine clearance (CCr) in BALB/c mice during i.p. and i.v. injection of ZnO NPs. Clearance was calculated based on urine volumes and serum and urine creatinine concentrations. *p < 0.05 versus 0 mg/kg. Figure S2. Histopathological analysis of kidneys after i.v. injection of ZnO NPs (2 mg/kg). Tissue sections were stained with H&E and observed microscopically. The black arrows indicate tubular dilatation. The black arrowheads indicate the loss of brush borders and flattened tubular epithelium. The asterisks indicate the reduction of Bowman’s space and the increase in cellularity in glomeruli. BS, bowman space; G, glomerulus. (DOCX 1351 kb) [file 12989_2016_163_MOESM1_ESM.docx]

**Additional file:**

| **Table S1. Biochemical tests including BUN, creatinine, GOT and**  **GPT after i.v. injection of ZnO NPs.** | | | | |
| --- | --- | --- | --- | --- |
| **Item/ Group** | **0 mg/kg** | **2 mg/kg** | **4 mg/kg** |  |
| **BUN (mg/dL)** | 15.78±1.35 | 18.94±2.17* | 18.88±2.24* |  |
| **Creatinine (mg/dL)** | 0.23±0.04 | 0.32±0.05* | 0.36±0.04* |  |
| **GOT (U/L)** | 90.26±15.05 | 125.52±26.49* | 150.62±12.82* |  |
| **GPT (U/L)** | 29.84±3.19 | 35.68±3.75* | 35.30±4.35* |  |

*p<0.05, ZnO NPs versus control.

**Figure S1**

Measurements of the creatinine clearance (CCr) in BALB/c mice during i.p. and i.v. injection of ZnO NPs. Clearance was calculated based on urine volumes and serum and urine creatinine concentrations. *p < 0.05 versus 0 mg/kg.


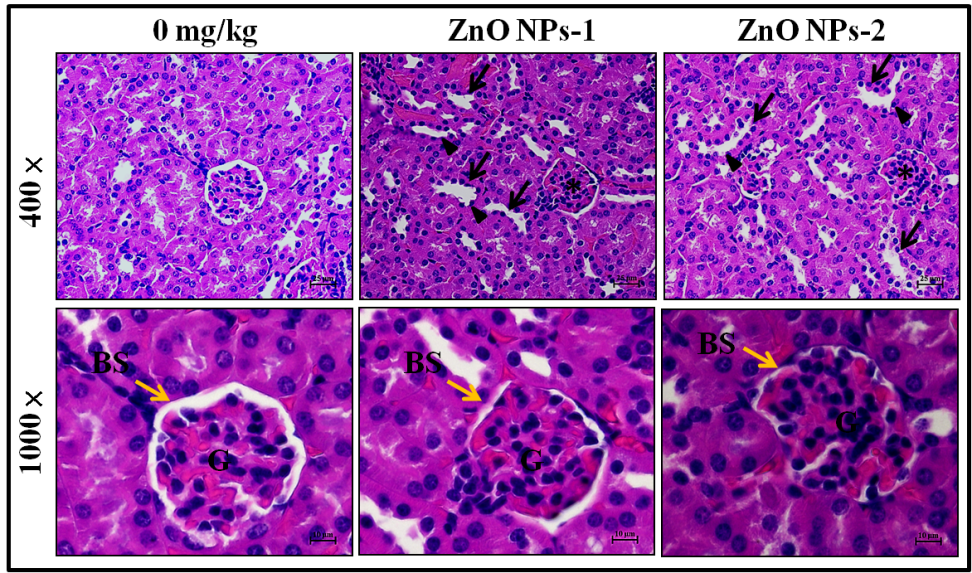


**Figure S2**

Histopathological analysis of kidneys after i.v. injection of ZnO NPs (2 mg/kg). Tissue sections were stained with H&E and observed microscopically. The *black arrows* indicate tubular dilatation. The *black arrowheads* indicate the loss of brush borders and flattened tubular epithelium. The *asterisks* indicate the reduction of Bowman’s space and the increase in cellularity in glomeruli. BS, bowman space; G, glomerulus.
